# Development of a Multi-Enzymatic Approach for the Modification of Biopolymers with Ferulic Acid

**DOI:** 10.3390/biom12070992

**Published:** 2022-07-17

**Authors:** Archontoula Giannakopoulou, Georgia Tsapara, Anastassios N. Troganis, Panagiota Koralli, Christos L. Chochos, Angeliki C. Polydera, Petros Katapodis, Nektaria-Marianthi Barkoula, Haralambos Stamatis

**Affiliations:** 1 Biotechnology Laboratory, Department of Biological Applications and Technologies, University of Ioannina, 45110 Ioannina, Greece; arxontoula.gian@gmail.com (A.G.); geo.tsapara@gmail.com (G.T.); apolyder@uoi.gr (A.C.P.); pkatapo@uoi.gr (P.K.); 2 Department of Biological Applications and Technologies, University of Ioannina, 45110 Ioannina, Greece; atrogani@uoi.gr; 3 Institute of Chemical Biology, National Hellenic Research Foundation, 48 Vassileos Constantinou Avenue, 11635 Athens, Greece; pkoralli@eie.gr (P.K.); chochos@eie.gr (C.L.C.); 4 Department of Materials Science & Engineering, University of Ioannina, 45110 Ioannina, Greece; nbarkoul@uoi.gr

**Keywords:** chitosan, cellulose, gelatin, feruloyl esterase, xylanase, laccase, ferulic acid, enzymatic cross-linking, antioxidant activity, antimicrobial activity

## Abstract

A series of polymers, including chitosan (CS), carboxymethylcellulose (CMC) and a chitosan–gelatin (CS–GEL) hybrid polymer, were functionalized with ferulic acid (FA) derived from the enzymatic treatment of arabinoxylan through the synergistic action of two enzymes, namely, xylanase and feruloyl esterase. Subsequently, the ferulic acid served as the substrate for laccase from *Agaricus bisporus* (AbL) in order to enzymatically functionalize the above-mentioned polymers. The successful grafting of the oxidized ferulic acid products onto the different polymers was confirmed through ultraviolet–visible (UV–Vis) spectroscopy, attenuated total reflectance (ATR) spectroscopy, scanning electron microscopy (SEM) and nuclear magnetic resonance (NMR) spectroscopy. Additionally, an enhancement of the antioxidant properties of the functionalized polymers was observed according to the DDPH and ABTS protocols. Finally, the modified polymers exhibited strong antimicrobial activity against bacterial populations of *Escherichia coli* BL21DE3 strain, suggesting their potential application in pharmaceutical, cosmeceutical and food industries.

## 1. Introduction

Over the last few years, extensive research efforts have been directed at producing high-value products and functional biomaterials derived from agro-industrial by-products. In particular, the utilization of lignocellulosic biomass, a feasible and inexpensive resource produced during agricultural processes, has attracted scientific interest for the production of biofuels and value-added products [1]. On the other hand, there is also a growing interest in exploiting natural polymers, such as chitosan or cellulose in diverse applications [2,3,4,5,6]. Among the various methods applied for the exploitation of natural resources, the utilization of enzymatic approaches has prevailed due to the increasing demand for environmentally friendly practices [7]. However, the complexity, as well as the heterogeneity of such proceedings, requires the synergistic action of several enzymes [8,9].

Cellulose consists of the most abundant polysaccharide, while chitosan derives from the deacetylation of chitin, the second most abundant polysaccharide on Earth [10]. The modification of chitosan aims at upgrading its functionality through the incorporation of bioactive compounds into its polysaccharide backbone [11,12,13]. Enzymatic cross-linking constitutes an attractive alternative approach to traditional chemical modifications for the development of advanced biomaterials with unique bioactive properties, usually superior to precursory polymers [14]. The advantages of enzymatic modification include, among others, the high specificity and the absence of hazardous chemicals and toxic processes. The enzymatic modification of chitosan with a wide variety of bioactive compounds, such as phenolics [15,16] or flavonoids [17,18], has been extensively studied. Apart from chitosan, the enzymatic modification of other polymers, such as cellulose [19] and hybrid hydrogels consisting of chitosan and gelatin [20,21], has also attracted scientific interest recently, especially for biomedical applications, ranging from wound healing to tissue engineering and drug delivery applications.

Feruloyl esterases (FEs) and xylanases are the principal enzymes that participate in the degradation of lignocellulosic biomass. More specifically, feruloyl esterases catalyze the cleavage of covalent ester bonds between hydroxycinnamic acids and polysaccharides, releasing ferulic acid and p-coumaric acid [22]. Ferulic acid is usually esterified at the C-5 hydroxy group of the arabinofuranosyl units of arabinoxylan. On the other hand, endo-xylanases catalyze the random cleavage of β-1,4-D-xylosidic linkages, generating xylo-oligosaccharides that could act as a favored substrate for releasing ferulic acid by FAs [23]. The synergistic action of a FE and an endo-xylanase could be combined in a biocatalytic process, as previously reported in the literature, in order to produce ferulic acid from lignocellulosic substrates, such as arabinoxylan [1,24]. Ferulic acid, the most abundant hydroxycinnamic acid, is characterized by strong antioxidant [25], antimicrobial [26,27] and anti-inflammatory [28] properties, and has attracted significant attention in the food and pharmaceutical industries. On the other hand, oxidoreductase enzymes, such as laccase [29,30] or tyrosinase [31], are ideal candidates for the enzymatic cross-linking of various bioactive compounds, such as the products derived by the laccase-catalyzed oxidation of ferulic acid and ethyl ferulate [15], onto polymers to enhance their biological properties. 

In the present study, the enzymatic activities of three biocatalysts, namely, feruloyl esterase from rumen microorganisms, endo-1,4-β-Xylanase from *Bacillus stearothermophilus* T6 and laccase from *Agaricus bisporus*, were combined for the enzymatic modification of different polymers. Firstly, the synergistic action of xylanase and feruloyl esterase was studied and optimized so that it could be successfully applied for the release of ferulic acid from insoluble wheat arabinoxylan (model substrate). Subsequently, we evaluated the cross-linking capacity of the laccase-oxidized ferulic acid products onto various biopolymers, including chitosan, carboxymethylcellulose (CMC) and a hybrid polymer consisting of chitosan and gelatin. The modified biopolymers were characterized by various spectroscopic methods such as ultraviolet–visible (UV–Vis) spectroscopy, attenuated total reflection (ATR) spectroscopy, scanning electron microscopy (SEM) and nuclear magnetic resonance (NMR) spectroscopy. Finally, the enzymatically modified polymers were characterized in terms of their antioxidant and antimicrobial activity, exhibiting a significant enhancement after their enzymatic modification. To the best of our knowledge, this is the first study to report the synergistic action of three enzymes for the exploitation of a ferulic acid-contained lignocellulosic substrate in order to develop functional biopolymers with enhanced biological activities. 

## 2. Materials and Methods

### 2.1. Materials

Endo-1,4-β-Xylanase from *Bacillus stearothermophilus* T6 (65 U/mg), feruloyl esterase from rumen microorganisms (300 U/mg) and wheat flour arabinoxylan (medium viscosity) were purchased from Megazyme (Bray, Co. Wicklow, Ireland). Chitosan from crab shells (low viscosity) and carboxymethyl cellulose, sodium salt, low viscosity (CMC) were purchased from Sigma-Aldrich (St. Louis, MO, USA). Gelatin from porcine skin was purchased from Fluka (Charlotte, NC, USA). Laccase from *Agaricus bisporus* (>4 U/mg, lyophilized), ethyl-4-hydroxy-3-methoxycinnamate (ethyl ferulate), 3,5-Dinitrosalicylic acid (DNSA), 1,1-diphenyl-2-picryl-hydrazyl (DPPH) and 2,2-azino-bis(3-ethylbenzthiazoline-6-sulfonic acid) (ABTS) were all purchased from Sigma-Aldrich (St. Louis, MO, USA).

All the other chemicals and reagents were of analytical grade and procured from reliable sources. Milli-Q water was used for the preparation of all the buffers and solutions.

### 2.2. Methods 

All experiments were performed in triplicate, and the values are given as mean values and experimental errors.

#### 2.2.1. Pre-Treatment of Arabinoxylan

The pre-treatment of arabinoxylan was based on Konieczna-Molenda et al., with slight modifications [32]. Briefly, 20 mg/mL of wheat arabinoxylan was dissolved in 25 mM phosphate buffer, pH 6.5, to a final volume of 5 mL. The arabinoxylan solution was incubated at 90 °C for 30 min at 1000 rpm. Then, the mixture was cooled for 30 min at room temperature and centrifuged at 4000 rpm for 10 min. The enzymatic reaction was directly performed in the supernatant collected.

#### 2.2.2. Enzymatic Treatment of Arabinoxylan

For the enzymatic treatment of arabinoxylan, enzymatic solutions containing xylanase and feruloyl esterase at a mass ratio of 1:5 were added into 20 mg/mL of pre-treated arabinoxylan, as previously described. The enzymatic reaction with a final volume of 5 mL was incubated at 70 °C, under constant stirring (750 rpm), for 24 h. Then, the reaction mixture was centrifuged at 4000 rpm for 10 min and the supernatant was collected. The mixture was eluted with methanol (1:1 ratio) and filtered with 0.45 mm filters. The identification and quantification of the produced ferulic acid were performed by high-performance liquid chromatography (HPLC) (Shimadzu, Tokyo, Japan) using a μBondapack C18 column, particle size 10 μm, length 300 mm, diameter 3.9 mm, and a diode array UV detector. Ambient conditions were used for analysis. The mobile phase consisted of acetonitrile (A) and 0.1% *v*/*v* acetic acid in water (B). The elution conditions applied for solvents A and B were as follows: 0–11 min 60% for solvent B and 40% for solvent A, 11–25 min 100% for solvent A and 25–30 min 60% for solvent B and 40% for solvent A. The elution conditions were performed at 35 °C with a flow rate of 1 mL/min, and the samples were detected at 280 nm. The injected volume was 40 µL and the UV absorption of the effluent was monitored at 280 nm. The retention time of ferulic acid was 3.6 min (Supplementary Materials, Figure S1). The quantification and characterization of ferulic acid were based on the calibration curve of ferulic acid under the same conditions.

#### 2.2.3. Determination of Enzymatic Activities

For the determination of feruloyl esterase activity, ethyl ferulate (EF) was used as substrate. The reaction (1 mL) was carried out in sodium phosphate buffer (25 mM, pH 6.5) that contained 0.5 mM EF. The reaction was initiated by the addition of a diluted enzyme solution. After 15 min incubation at 40 °C (750 rpm), the enzymatic activity was terminated by incubation at 100 °C for 5 min. The reaction mixture was eluted with methanol (1:1 ratio) and filtered with 0.45 mm filters. The quantification of ferulic acid was performed by high-performance liquid chromatography (HPLC) (Shimadzu, Tokyo, Japan), as mentioned above. One unit of enzyme activity was defined as the amount of enzyme that released 1 μmol of ferulic acid per min under standard conditions.

For the determination of xylanase activity, 5 mg/mL of insoluble wheat arabinoxylan (WAX) was used as a substrate. The reaction mixture consisted of 400 μL of 1.0% (*w*/*v*) substrate dissolved in 100 mM acetate buffer (pH 5.0) and 100 μL of the diluted enzyme. After 15 min incubation at 70 °C (750 rpm), the enzymatic activity was terminated by incubation at 100 °C for 5 min, followed by centrifugation at 12,000 rpm for 5 min. The concentration of reducing sugars was determined using the 3,5-dinitrosalicylic acid (DNS) method [33]. Briefly, 250 µL of the sample was mixed with 250 µL of DNSA, followed by boiling for 5 min. One unit of enzyme activity was defined as the amount of enzyme that released 1 μmol of reducing sugars per min. Reducing sugars were estimated using a xylose standard curve. 

The determination of laccase activity was based on the oxidation of a standard substrate, namely, ABTS. The enzymatic activity of laccase using ABTS as substrate was determined through the measurement of the increase in absorbance during the oxidation reaction of the ABTS. The absorbance was measured with an Elisa plate Reader (Elisa Infinite F50) at 405 nm. Absorption values were taken every 1 min for a total of 10 min, at 25 °C. The reaction (1 mL) was carried out in acetate buffer (100 mM, pH 4.58) that contained 1 mM ABTS, and it was initiated by the addition of 12 μg/mL of enzyme solution. One unit of enzyme activity was defined as the amount of enzyme required to oxidize 1 μmol of ABTS per min. 

#### 2.2.4. Optimization of the Production of Ferulic Acid

In order for the maximum production of ferulic acid to be achieved by the bi-enzymatic system, several parameters, such as the reaction time, the reaction temperature and the mass ratio of xylanase to esterase, as well as the effect of substrate concentration, were evaluated and optimized by one factor at a time. To determine the effect of the reaction time on the production of ferulic acid, an enzyme mixture solution consisting of xylanase and esterase (at a mass ratio of 1:5) was added to 20 mg/mL of pre-treated arabinoxylan (as previously described) in 25 mM phosphate buffer, pH 6.5, at 70 °C, under constant magnetic stirring (750 rpm). At standard time intervals, a certain amount was collected and analyzed through HPLC to determine the amount of ferulic acid produced, as described before. The same procedure was also repeated at 40 °C and 50 °C in order to evaluate the effect of the temperature reaction on the amount of ferulic acid produced. The effect of the mass ratio of xylanase to esterase was determined by performing the previously described reaction at varying xylanase–esterase ratios (3:5, 3:2, 3:1, 1:10 and 1:20), that were selected based on preliminary results. Finally, the effect of the substrate concentration was evaluated by adding the enzyme solution containing both xylanase and esterase (at a mass ratio of 1:5) at different arabinoxylan concentrations (1, 2.5, 5, 10, 15, 20 and 25 mg/mL) in 25 mM phosphate buffer, pH 6.5, at 70 °C, and the amount of ferulic acid produced was determined as described above.

#### 2.2.5. Synergy Studies

In order to investigate the existence of synergistic or competitive interactions between the two enzymes, their simultaneous or successive addition in the reaction mixture was tested and evaluated in terms of the ferulic acid produced. The reaction involving the simultaneous addition of the enzymes has been previously described. In order to study the successive addition of the enzymes, an enzymatic solution containing only xylanase was firstly added to 20 mg/mL of arabinoxylan in 25 mM phosphate buffer, pH 6.5. The reaction was incubated at 70 °C, 750 rpm, for 30 min. Then, esterase was added, while the temperature was lowered to 40 °C so as to ensure the optimal reaction conditions for each enzyme. The reaction was incubated for 24 h under constant stirring (750 rpm) and the amount of ferulic acid produced in both cases was determined through HPLC analysis.

#### 2.2.6. Purification of Chitosan and Preparation of the CS Solution

For the purification and activation of the chitosan, initially, a chitosan solution of 1.5% *w*/*v* was prepared by dissolving the chitosan flakes (1.5 g) into an aqueous acetic acid solution (1% *v*/*v*). The chitosan–acetic acid mixture was then incubated O/N at 70 °C under constant stirring (750 rpm). After the O/N incubation, the solution was centrifuged for 15 min at 4000 rpm. The resulting insoluble material was removed and the supernatant was stored. The pH of the supernatant was adjusted to pH 4.58 by adding NaOH (2 M).

#### 2.2.7. Preparation of the CS–GEL Solution

For the preparation of chitosan–gelatin solution, initially, a 2% *w*/*v* chitosan solution and a 2% *w*/*v* gelatin solution were prepared. For the preparation of gelatin solution, 2 g of gelatin was dissolved in 100 mL of 100 mM acetate buffer, pH 4.58. The solution was left at 60 °C, under constant stirring (750 rpm), until complete dissolution. For the preparation of the chitosan solution, 2 g of chitosan flakes were added slowly into 100 mL of 1% *v*/*v* hydrochloric acid solution at room temperature until total dissolution. Then, the pH of the prepared solution was adjusted to 4.58 with the addition of 2 M NaOH solution. Finally, the prepared chitosan and gelatin solutions were mixed at a ratio of 2:3 *w*/*w* at 25 °C, overnight, under constant stirring (750 rpm). The resulting chitosan–gelatin solution was stored at 4 °C for further use.

#### 2.2.8. Preparation of the CMC Solution

For the preparation of the CMC solution, 2 g of CMC powder was dissolved in 100 mL of 100 mM acetate buffer, pH 4.58. The solution was stirred under constant stirring (750 rpm) at 25 °C, until total dissolution was achieved.

#### 2.2.9. Enzymatic Oxidation of FA and Its Grafting onto the CS, CS–GEL and CMC Films

The prepared CS, CS–GEL and CMC solutions were enzymatically modified by laccase from *Agaricus bisporus* with ferulic acid. Specifically, to a final reaction volume of 20 mL, 1.3% *w*/*v* CS or 1.3% *w*/*v* CS–GEL or 1.3% *w*/*v* CMC solution was mixed with 5 mM ferulic acid solution (solubilized in MeOH, or 0.5 mM ferulic acid produced by the enzymatic treatment of arabinoxylan), and 0.086 U/mL (60 μg/mL) laccase from *Agaricus bisporus* (solubilized in 100 mM acetate buffer, pH 4.58) was added to initiate the reaction. The reactions were plated on Petri dishes and incubated at 30 °C and 80 rpm O/N. At standard time intervals, 1 mL of the reaction was taken in order to monitor the reaction progress through the changes observed in the UV spectra, in the range of 200–400 nm. Additionally, all the control reactions were performed (CS, CS–GEL and CMC with ferulic acid in the absence of laccase) and their UV spectra were recorded at standard time intervals. After the O/N incubation, the plates were then left to dry at 45 °C O/N to form films. All the resulting films were rinsed several times with methanol and once with ultra-pure water to remove any unbound compounds.

#### 2.2.10. Characterization of the Biopolymers and Their Enzymatically Functionalized Derivatives

##### Ultraviolet (UV)–Visible Spectroscopy

For the characterization of the prepared films, an ultraviolet–visible (UV–Vis) spectrum in the range of 200–800 nm was obtained in a quartz cell with a path length of 1 cm. For the preparation of the samples, about 1 mg of each film (modified and non-modified) was dissolved in 1% *v*/*v* aqueous acetic acid solution. In addition, a ferulic acid sample with a final concentration of 0.5 mM was prepared. Acetic acid solution was used as a control.

##### Attenuated Total Reflection (ATR) Spectroscopy

The prepared films were additionally characterized by infrared spectroscopy with a Jasco FT/IR 4700 attenuated total reflection (ATR) technique. The spectra were recorded in the range of 400–4000 cm^−1^, while the final spectrum was obtained from the mean value of 128 scans with a resolution of 2 cm^−1^.

##### Scanning Electron Microscopy (SEM)

Scanning electron microscopy (SEM) was applied for the characterization of both the enzymatically modified biopolymers and the non-modified films regarding their surface morphology. SEM measurements were performed on the JEOL JSM-7610FPlus device, which integrates a full set of detectors for secondary electrons and backscattered electrons. Before analysis, the samples were deposited on a precleaned gold (Au) cylindrical surface as films pretreated with gold via a sputtering process. Then, the samples were inserted in a vacuum chamber with 9.6 × 10^−5^ bar pressure and visualized via SEM.

##### Nuclear Magnetic Resonance (NMR) Spectroscopy

For the NMR analysis, a standard sample preparation was followed according to Chen et al. with some modifications [34]. Initially, the enzymatic reactions were performed with a final chitosan concentration of 0.15% *w*/*v*. The reactions were incubated overnight and, after incubation, the modified chitosan was precipitated by increasing the pH of the reaction to 7. This increase was achieved by adding NaOH (2 M), and the solutions were subsequently centrifuged in duplicate at 10,000 rpm for 10 min. The resulting precipitate was rinsed several times with MeOH and finally with deionized water. The purified polymer was dissolved at a low pH (pH = 1.5 to 2) and lyophilized. After lyophilization, the precipitate was re-dissolved in 3 mL of deuterated water (99.9% D_2_O) and lyophilized again. The procedure of redissolving and reprecipitating was repeated two additional times to remove any impurities. Finally, for the ^1^H-NMR analysis, the purified polymer was dissolved in 3 mL of deuterated water and the pH was lowered to 1–2 using deuterated HCL. The same procedure was followed for the non-modified chitosan.

The NMR spectra were recorded on a Bruker AVANCE 500 MHz spectrometer at 313 K, using H_2_O–D_2_O as solvent. The concentration of the samples was 1.5 mg/mL, approximately. Routine parameters were used to record the one-dimensional spectra. One dimensional spectra were recorded with a sweep width of 4100 Hz and 32 K (zero-filled to 64 K) data points. The chemical shifts are reported with respect to the resonance of TMSP, which was used as an internal standard. The WATERGATE-5 pulse sequence was used for the suppression of the signal of the water. Data were processed using TopSpin 3.1 standard software. An exponential weight function was used prior to Fourier transformation.

#### 2.2.11. Estimation of the Grafting Efficiency

Folin–Ciocalteu reagent was used to determine the amount of total phenolics bound onto the biopolymers. Briefly, an aliquot of each film was washed five times with 500 μL of methanol to remove any phenolic acids (FA) not bound onto biopolymers and each resulting fraction was collected (sample). To a final reaction volume of 2 mL, 40 μL of each sample was added along with 100 μL of Folin–Ciocalteu reagent and 960 μL of ultra-pure water. After 3 min, 200 μL of Na_2_CO_3_ solution (20% *w*/*v*) was added and the volume was completed with ultra-pure water (700 μL). The reactions were left for 1 h in a dark place and then the absorbance of the samples was measured at 725 nm. Additionally, a standard curve of ferulic acid was prepared according to the reaction described above, with a concentration range of 0.156 mM to 5 mM. The resulting equation was: y = 0.30[FA] + 0.02, where y corresponds to the absorption value at 725 nm.

#### 2.2.12. Evaluation of the Antioxidant Activity of the Prepared Films and Their Derivatives

##### Antioxidant Activity According to the Oxidized ABTS Protocol

The determination of the antioxidant activity of modified and non-modified biopolymer films was performed according to the oxidized ABTS protocol (ABTS•+). Initially, a solution of oxidized ABTS (ABTS•+) was prepared by the reaction of 7 mM ABTS (dissolved in ultra-pure water) with 2.45 mM potassium persulfate. The solution was incubated in a dark place for 18 h at room temperature. Prior to use, the oxidized ABTS solution was diluted with phosphate solution to give an absorbance of 0.700 ± 0.025 at 734 nm. To determine the antioxidant activity, about 1.0 mg of each film and 1 mL of the oxidized ABTS solution were mixed in an Eppendorf tube. At standard time intervals (0, 5, 10, 20, 30 and 40 min), 300 μL of the reaction was taken and absorbance was measured at 734 nm with an Elisa plate reader. After each measurement, the sample was returned to the Eppendorf tube. The results were expressed as percentages of antioxidant activity as a function of incubation time. The percentage antioxidant activity was calculated according to the equation:

Antioxidant activity (%) = [(A_control_ − A_sample_)/A_control_] × 100

where A_control_ is the absorption of oxidized ABTS after dilution with phosphate solution (PBS).

##### Antioxidant Activity According to the DPPH Protocol

The free radical scavenging activity of the modified and non-modified films was measured according to the methodology described by Blois [35], in which the bleaching rate of a stable free radical, DPPH•, is monitored at a characteristic wavelength in the presence of the sample. Briefly, 0.1 mM of DPPH• solution in ethanol was prepared and 300 μL of this solution was added into different amounts (mg) of the samples. Then, 700 μL of ethanol was added to reach a final volume of 1 mL. After 30 min, the absorbance was measured at 517 nm. A lower absorbance of the reaction mixture indicates higher free radical scavenging activity. The capability of the functionalized polymers to scavenge the DPPH• radicals was calculated using the equation:

Antioxidant activity (%) = [(A_control_ − A_sample_)/A_control_] × 100

where A_control_ is the absorbance of control sample and A_sample_ is the absorbance of the grafted polymer.

#### 2.2.13. Evaluation of the Antimicrobial Activity of the Prepared Films and Their Derivatives

For the determination of the antimicrobial activity of the prepared films (modified and non-modified), initially, 100 μL of bacterial population was inoculated from stock bacterial populations of *E. coli* BL21DE3 strain, stored in glycerol, in 5 mL of fresh Lysogeny Broth (LB). The preculture was incubated at 37 °C, O/N, under constant stirring at 180 rpm. After the O/N incubation, the pre-culture absorbance was measured at 600 nm and diluted with fresh LB so that the new culture reached an OD of about 0.08 absorbance. The new culture was incubated at 37 °C, under stirring at 180 rpm, for about 2 h, until the bacterial population passed the exponential phase and presented an optical density (O.D.) of 0.2–0.5. The culture was then centrifuged at 4000 rpm for 5 min. The resulting supernatant was discarded and the bacterial precipitate was redissolved in saline (0.9% *w*/*v* NaCl). Then, three successive washes of the bacterial pellet with saline followed and finally the pellet was redissolved in an equal volume of the culture. Bacterial population samples of 10^7^ CFU/mL were prepared. Approximately 1 mg of each film was added into an Eppendorf tube containing 100 μL of the bacterial population which was then incubated for 12 h in a cold chamber. Then, 25 μL of the bacterial population that interacted with the films were inoculated into 225 μL of fresh LB medium in an Elisa microplate well. The microplate was then placed in an incubator chamber at 37 °C and measurements of the O.D. at 600 nm were taken each hour for a total of eight hours. Finally, a growth curve of the bacterial population, both in the presence and in the absence of the films, was prepared.

## 3. Results and Discussion

### 3.1. Determination of the Enzymes’ Specific Activities

The specific activities of the enzymes were determined as described in Section 2.2.3. The specific activity of xylanase using arabinoxylan as substrate was found to be 65 U/mg, while the specific activity of feruloyl esterase with ethyl ferulate as substrate was defined as 300 U/mg. The specific activity of laccase AbL with ABTS was determined as 14 U/mg.

### 3.2. Optimization of the Bi-Enzymatic System on the Production of Ferulic Acid

In order for the maximum ferulic acid production to be achieved by the synergistic action of xylanase and feruloyl esterase, several parameters regarding the reaction conditions, such as the reaction time, the temperature, the arabinoxylan concentration as well as the xylanase to esterase mass ratio, were examined and optimized, one factor at a time.

**Figure 1:**
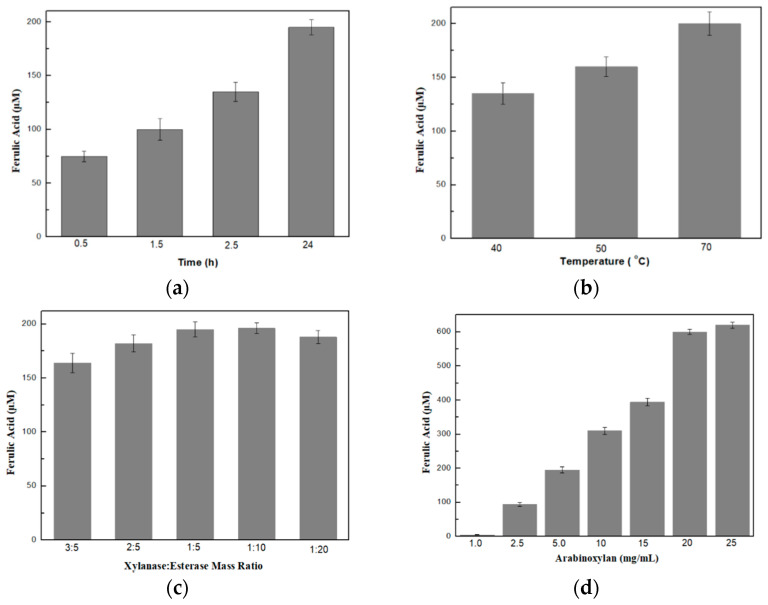
Effects of different parameters on the amount of ferulic acid produced: (**a**) reaction times; (**b**) temperatures of reactions; (**c**) xylanase–esterase mass ratios; and (**d**) substrate concentrations.

Regarding the reaction time, as shown in Figure 1a, maximum ferulic acid production (about 200 μΜ) was observed after 24 h. At 30 min, only about 75 μΜ of ferulic acid was produced, gradually increasing over time, suggesting that an overnight incubation was necessary in order to attain the maximum possible production of ferulic acid. Ferulic acid production was also estimated for 48 h but no further increase was detected, probably due to the reduced operational stability of the enzymes at high temperature under continuous operation. For this reason, the reaction stopped at 24 h, where maximum ferulic acid production was detected. 

Regarding the effect of reaction temperature on the production of ferulic acid, 70 °C seems to be a more effective temperature for obtaining the maximum production of ferulic acid in comparison to 50 °C or 40 °C, as shown in Figure 1b. This could be probably attributed to the fact that endo-xylanase, a highly thermostable enzyme, catalyzes the breakage of arabinoxylan optimally at 70 °C. Then, the generated xylo-oligosaccharides could be more efficiently catalyzed by FE. Furthermore, in order to ensure the optimal reaction conditions for both enzymes, the production of ferulic acid was evaluated in the temperature range of 40–70 °C and not at temperatures higher than 70 °C due to the deactivation of the feruloyl esterase enzyme under these conditions (data not shown).

Further, by maintaining the reaction temperature at 70 °C and the reaction time at 24 h, we examined the effect of the xylanase-to-esterase mass ratio on the production of ferulic acid. As shown in Figure 1c, the 1:5 and 1:10 xylanase–esterase mass ratios rendered the highest amount of ferulic acid. Finally, we evaluated the effect of arabinoxylan concentration on the production of ferulic acid. As shown in Figure 1d, the concentration of ferulic acid produced increased as arabinoxylan concentration increased, reaching a maximum concentration (about 0.6 mΜ) with 25 mg/mL arabinoxylan. However, after reaching a maximum value, no further increase occurred, probably due to inhibition phenomena by the substrate, suggesting that the system reaches its maximum productivity at an arabinoxylan concentration of 25 mg/mL.

According to these results, the highest amount of ferulic acid can be achieved at 70 °C, at a mass ratio of xylanase to esterase of 1:5, at an arabinoxylan concentration of 25 mg/mL and with overnight incubation of the reaction. Under these conditions, the enzymatic treatment of arabinoxylan renders more than 0.5 mM of ferulic acid.

### 3.3. Synergy Studies

**Figure 2:**
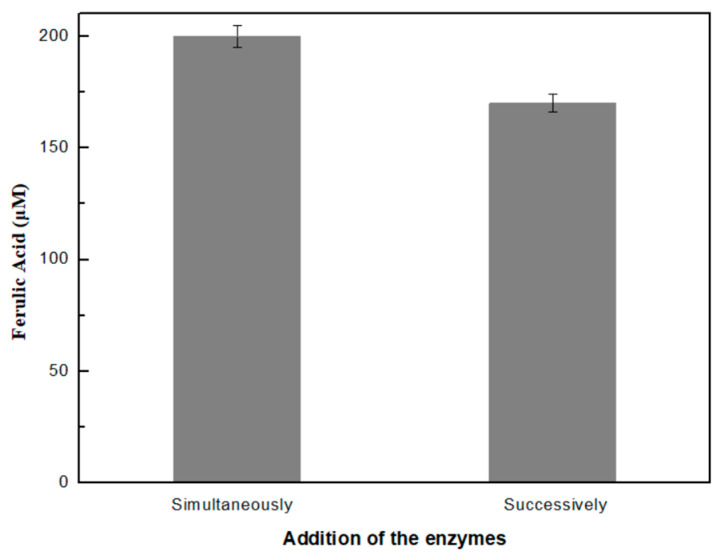
Effect of the simultaneous and successive addition of xylanase and esterase on the amount of ferulic acid produced.

In order to investigate the existence of any competitive effects between the enzymes on the enzymatic treatment of arabinoxylan, we tested either their simultaneous or their successive addition into the reaction mixture in terms of FA produced. For this reason, an enzyme cocktail consisting of xylanase and esterase at a mass ratio of 1:5 was added into 20 mg/mL of pre-treated arabinoxylan. The reaction was carried out at 70 °C for 24 h. The same mass ratio was applied when the enzymes were added successively into the reaction mixture. As shown in Figure 2, a higher amount of ferulic acid was produced when the two enzymes were added simultaneously in the reaction. This result probably indicates that the two enzymes act synergistically in the co-production of ferulic acid from arabinoxylan. Initially, the pre-treatment of arabinoxylan resulted in the disruption of the close inter-component linkages between the constituents of arabinoxylan, producing a preferred substrate for xylanase for the cleavage of β-1,4-D-xylosidic linkages. Esterase acts directly on the produced xylo-oligosaccharides to cleave ester bonds, releasing ferulic acid. It seems that the simultaneous existence of esterase in the reaction mixture renders a higher amount of ferulic acid in comparison to the ferulic acid produced when esterase is added in a following step. 

### 3.4. Preparation of Biopolymer Films

**Figure 3:**
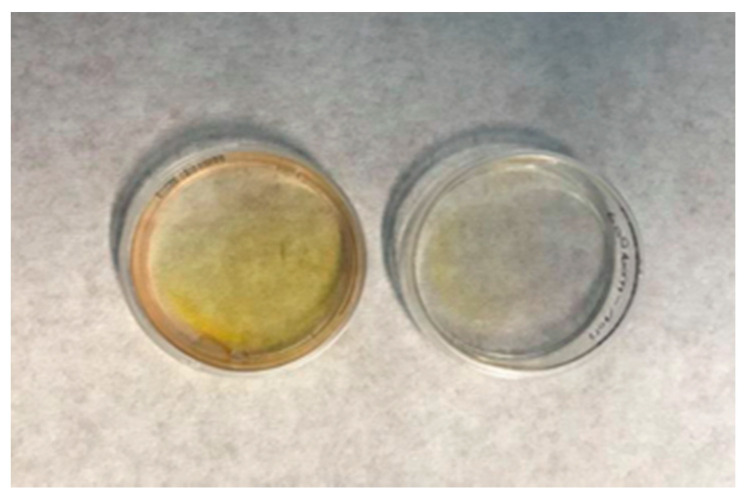
Illustration of the enzymatically modified (left) and non-modified (right) biopolymer membranes.

The first evidence that the CS, CS–GEL, and CMC films could be successfully modified by the laccase oxidation of ferulic acid was the visual observation that the reaction solutions turned yellow during the course of the reaction while the control solutions remained colorless. More specifically, as shown in Figure 3, the enzymatically modified membranes were characterized by a yellow-orange color, while no color changes were observed in the control films, containing either the polymer with laccase or the polymer with ferulic acid. Similar observations have been reported for the enzymatic modification of cellulose and chitosan films with chlorogenic acid [36], as well as in the preparation of ferulic acid-grafted chitosan using a recombinant bacterial laccase, where the produced derivatives were characterized by a stable orange color [12].

### 3.5. Enzymatic Oxidation of FA and Its Grafting onto the Biopolymers

**Figure 4:**
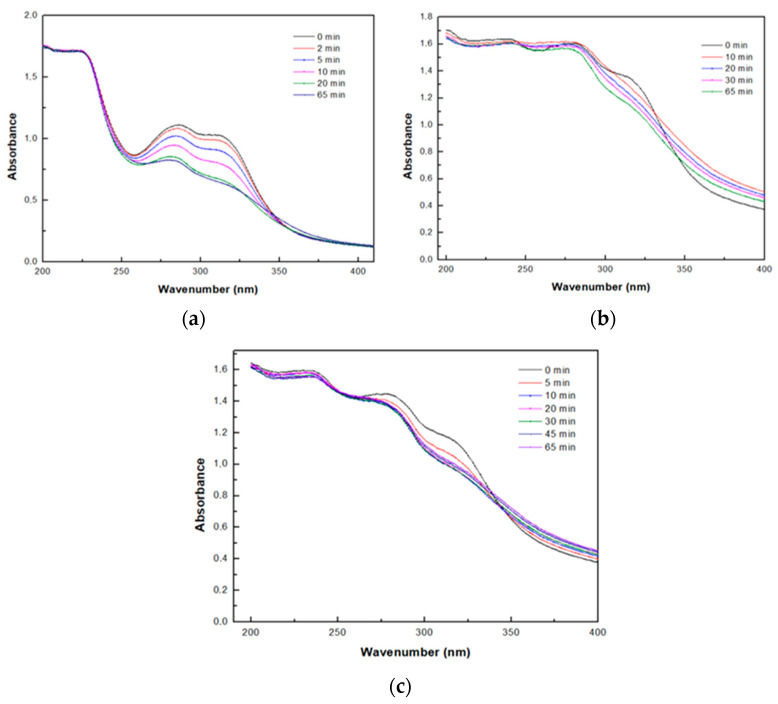
Changes in the UV spectra of the ferulic acid oxidation by laccase from *Agaricus bisporus* in the presence of: (**a**) chitosan; (**b**) chitosan–gelatin; and (**c**) CMC solutions.

To provide spectroscopic evidence that laccase mediates the successful oxidation of ferulic acid to reactive products that subsequently graft onto the different polymers, we monitored the changes in the UV spectra during the enzymatic oxidation of FA, as shown in Figure 4. In the case of chitosan, a significant decrease in the absorption band in the range of 275–320 nm was observed over time, followed by its gradual shift to 280 nm (Figure 4a). Concerning the enzymatic oxidation of ferulic acid in the CS–GEL and CMC solutions, only a slight decrease in certain peaks in the range of 250–325 nm was observed in both cases (Figure 4b,c). Furthermore, in all cases, a color change from non-colored to orange was observed immediately after the addition of the enzyme, as described in Section 3.4. For all biopolymers used, the corresponding control experiments were performed in the absence of laccase (Supplementary Materials, Figure S2) and no spectral or color changes were observed, indicating that the presence of the enzyme is essential for the oxidation of ferulic acid and the subsequent grafting to the biopolymers.

Thus, the overall changes in the UV spectra, mainly characterized by a reduction in the maximum absorbance during the incubation of the enzymatic reactions constituted the first indication of the successful ferulic acid oxidation and the subsequent modification of the polymers. Similar changes in the UV spectra have been also previously reported for the enzymatic modification of polymers with phenolic compounds [37].

### 3.6. Structural Characterization of the Biopolymers and Their Enzymatically Functionalized Derivatives

#### 3.6.1. Ultraviolet (UV)–Visible Spectroscopy 

**Figure 5:**
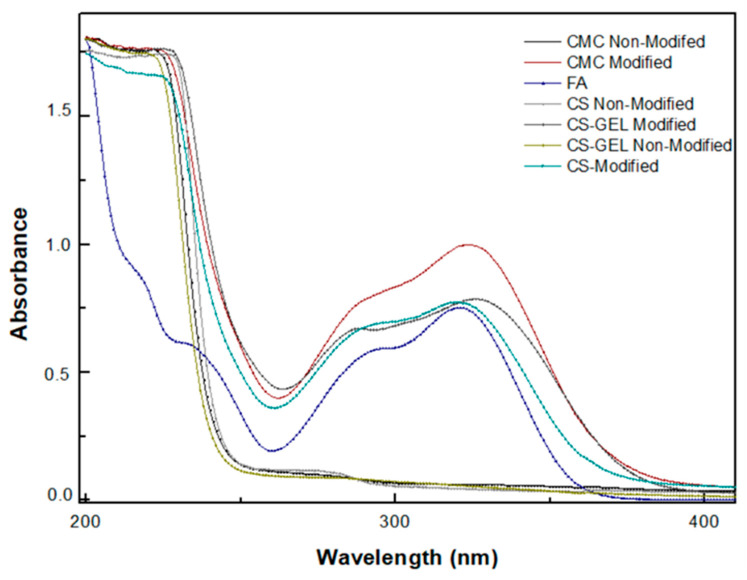
Ultraviolet-visible (UV–Vis) spectra in the range of 200–400 nm of enzymatically modified and non-modified biopolymer membranes dissolved in 1% *v*/*v* aqueous acetic acid solution, as well as a ferulic acid (FA) sample with a final concentration of 0.5 mM. Acetic acid solution was used as a control.

To provide further evidence that the products of the laccase-catalyzed oxidation of ferulic acid were covalently bound to the different biopolymers, we measured the UV spectra of the prepared films and referenced them against the absorbances of the same films without any enzymatic treatment. As shown in Figure 5, CS, CMC and CS–GEL films did not show distinct peaks in the range of 200–400 nm, in contrast to ferulic acid, which strongly absorbed in the range of 250–350 nm. However, when CS, CMC and CS–GEL derivatives were enzymatically modified with laccase, their spectrum showed a large increase in the absorbance between 300 and 350 nm, suggesting the successful oxidation of FA by laccase. The phenoxy radicals that resulted from the FA oxidation were delocalized and the radical intermediates were stabilized by C–C or C–O coupling reactions. Then, the reactive quinone methides formed in the dimerization coupling process could be stabilized by reacting with the free –OH and –NH_2_ groups of the biopolymers, rendering a strong increase in their UV spectra [38]. Furthermore, for each polymer studied, the corresponding control experiments were performed in the absence of laccase (data not shown) and no spectral changes were observed, indicating that the presence of the enzyme is necessary for the oxidation of ferulic acid and subsequent modification of the polymers. These results suggest that the reactive products synthesized by the laccase-catalyzed oxidation of ferulic acid could be covalently grafted onto the CS, CMC, and CS–GEL derivatives, as reported in other studies [12].

#### 3.6.2. Attenuated Total Reflection (ATR) Spectroscopy

**Figure 6:**
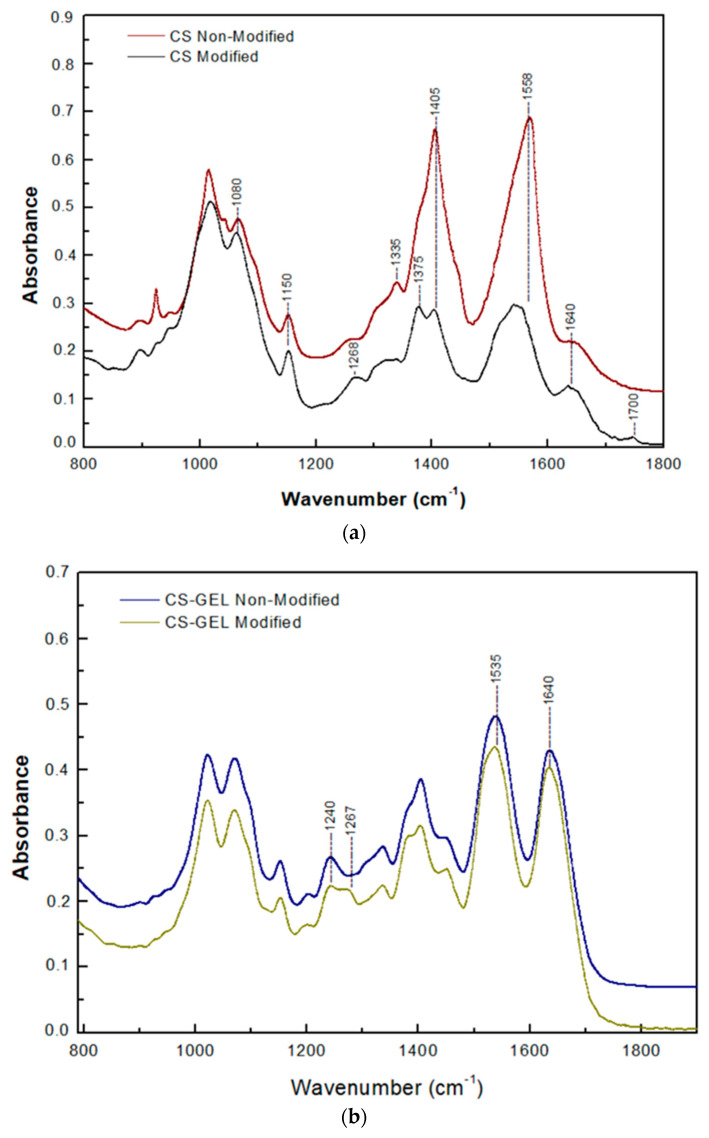

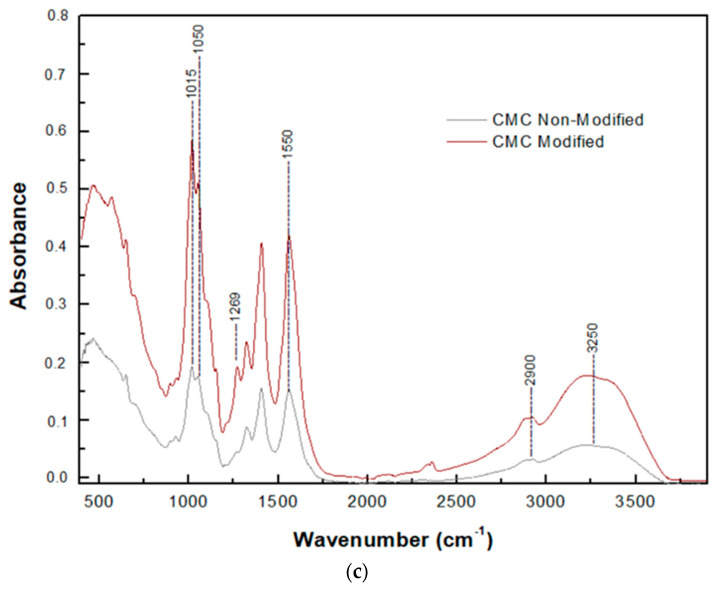
Infrared (ATR) spectra of chitosan (**a**), chitosan/gelatin (**b**) and CMC membranes (**c**) before and after their enzymatic modification with ferulic acid by laccase from *Agaricus bisporus*.

Another approach for the structural characterization of the products grafting onto the biopolymers is the ATR spectra measurement of the non-modified and enzymatically modified CS, CMC, and CS–GEL derivatives, as presented in Figure 6. Regarding the CS spectrum, as shown in Figure 6a, both chitosan films displayed all the peaks mentioned in the literature as characteristic for this polysaccharide, namely, the peak at 1640 cm^−1^, corresponding to the Amide I region (C=O vibrations), as well as the peak at 1558 cm^−1^ that corresponds to the Amide II region (NH bending vibrations) [39]. Additionally, the distinct peaks, present in both spectra, namely, the absorption band at around 1150 cm^−1^ (anti-symmetric stretching of C–O–C bridges) and the peak at around 1080 cm^−1^ (skeletal vibration involving C–O stretching), are characteristic of the chitosan saccharide structure, according to the literature [40]. The spectra of the modified chitosan film were characterized by the absence of the peak at 1335 cm^−1^, which is a peak attributed to the representative amine groups of the chitosan [15], suggesting a loss of NH_3_ groups [13]. Furthermore, a new band appeared at around 1700 cm^−1^ in the spectrum of the modified CS, corresponding to the ester band [11], while the reduction in absorption at 1404 cm^−1^ is noticeable, this being attributed to the bending vibrations of CH_2_, a group adjacent to the hydroxyl group of the C6 carbon atom. However, peaks in the wider range of 1260–1230 cm^−1^ were attributed to C–C(O)–C amplitude vibrations, a bond that occurs in esters. The changes detected in the spectrum of modified chitosan imply that both its amino and hydroxyl groups were subjected to covalent binding by the reactive products of FA oxidation. As previously mentioned, the resulted radical intermediates in FA oxidation can be stabilized through C–C or C–O coupling reactions, forming reactive quinone methides that can be further stabilized by reacting with both the free –OH and –NH_2_ groups present in CS [38].

Regarding the CS–GEL spectra, as shown in Figure 6b, both samples (modified and non-modified) presented the characteristic bands at 1640 cm^−1^ (Amide I) and 1535 cm^−1^ (-NH_2_ bending). However, in the spectrum of the enzymatically modified CS–GEL membrane, the absorbance of these peaks decreased compared to the non-modified membrane, probably suggesting the enrollment of these groups in the cross-link reaction. Additionally, in the modified membrane a new peak appeared in the region of 1220–1275 cm^−1^ at around 1267 cm^−1^, which was attributed to C–N stretching of aryl amides, whereas the peak at 1240 cm^−1^ could be attributed to the decrease in alkyl amines [21]. These changes also confirm the successful modification of the free amino groups present in the CS–GEL hybrid polymer by the reactive products of the FA oxidation. 

Concerning the CMC spectra, as shown in Figure 6c, both spectra (modified and non-modified CMC) presented the characteristic peaks at 3250 cm^−1^, 1550 cm^−1^ and 1050 cm^−1^, assigned to OH stretching, C=O, and –O– stretching of ether linkage, respectively. However, a new peak appeared at 1269 cm^−1^ in the spectrum of the enzymatically modified CMC, while this peak was undetectable in the spectrum of the non-modified CMC. Furthermore, the wide peak at around 1015 cm^−1^ considerably increased in the CMC modified spectrum, probably suggesting an ether bond linkage [19,41]. Furthermore, an increase in the intensity of the peak at 2900 cm^−1^ appeared in the spectrum of modified CMC and could probably be attributed to the C–H of methyl (–CH_3_) and methylene (–CH_2_) phenolic groups [12]. These results confirm the successful binding of the laccase-oxidized FA reactive products onto CMC, probably through the formation of ester bonds.

#### 3.6.3. Scanning Electron Microscopy (SEM)

**Figure 7:**
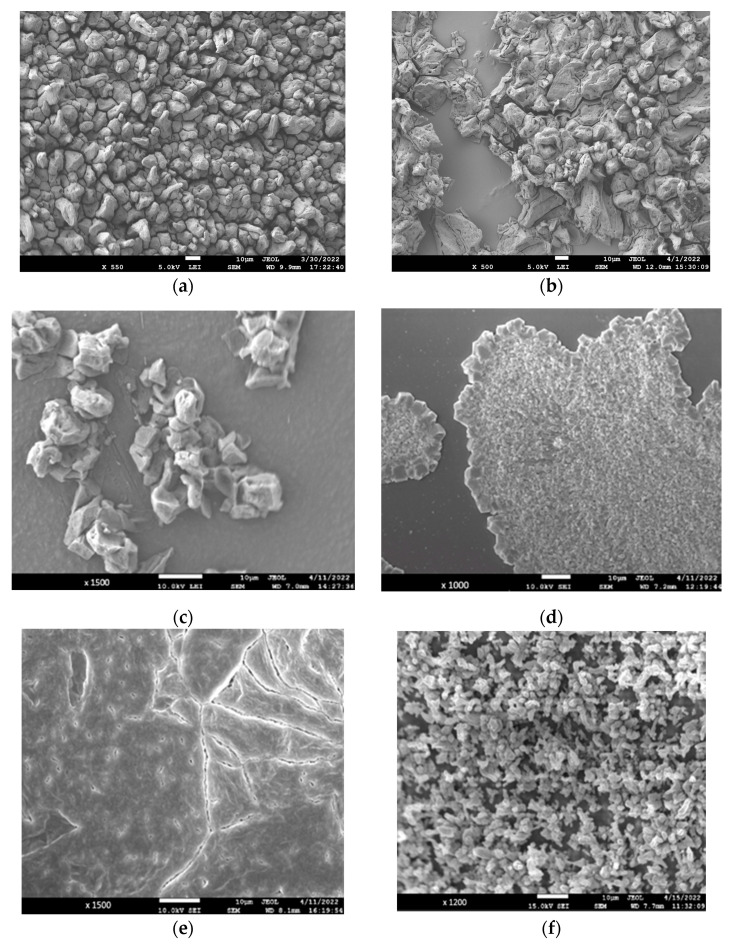
SEM micrographs of the surfaces of films based on CS (**a**), FA-CS derivative (**b**), CS–GEL (**c**), CS–GEL-FA derivative (**d**), CMC (**e**) and CMC-FA-derivative (**f**) films.

The surface morphology of CS, CS–GEL, CMC and their enzymatically modified derivatives films was studied through SEM, and the corresponding SEM micrographs are represented in Figure 7. As shown, the non-modified CS film (Figure 7a) presents a continuous and relatively smooth surface. However, after the enzymatic grafting of the FA derivatives onto chitosan, both the roughness and heterogeneity of its surface significantly increased, as depicted in Figure 7b, probably due to the high quantity of FA products grafted, as reported above. Concerning the SEM images of the CS–GEL films, as shown in Figure 7c, the non-modified CS–GEL film presents a comparatively smoother surface than the enzymatically modified CS–GEL. The oxidation of FA and the subsequent grafting of the ferulic acid derivatives onto the CS–GEL film altered the surface morphology, as shown in Figure 7d, resulting in a more irregular, uneven, rough structure. Finally, the SEM micrographs of the non-modified and enzymatically modified CMC films are shown in Figure 7e,f, respectively. Similarly, it was observed that the FA-modified CMC film presented a more heterogeneous and rougher structure than the control film. These findings also support the successful grafting of the FA oxidative derivatives onto the various biopolymers. Similar changes in the surface morphology have also been reported for the enzymatic modification of chitosan with various cinnamic acids [11], for quercetin-based chitosan–gelatin films [20] as well as for carboxymethylcellulose films modified with phenol compounds [41].

#### 3.6.4. Nuclear Magnetic Resonance (NMR) Spectroscopy

**Figure 8:**
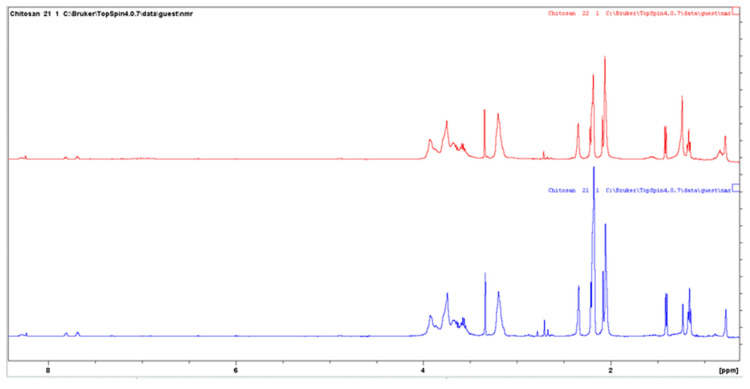
Solution phase ^1^H NMR spectra of chitosan (blue) and enzymatically modified chitosan (red). Both samples had the same chitosan concentration (~1.5 mg/mL).

**Figure 9:**
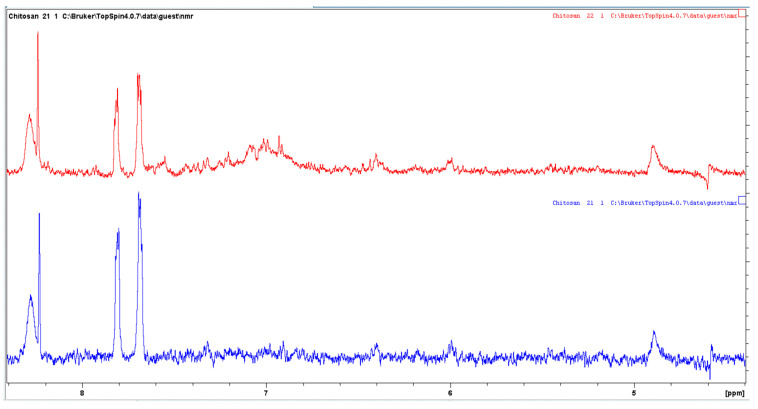
Enhanced solution phase ^1^H NMR spectra of chitosan (blue) and enzymatically modified chitosan (red). Both samples had the same chitosan concentration (~1.5 mg/mL).

In order to support the evidence that chitosan was successfully modified, we also applied ^1^H-NMR. For this reason, untreated and enzymatically modified chitosan solutions were prepared as previously described in the experimental section. Figure 8 shows the ^1^H-NMR spectra of a mixture containing chitosan and ferulic acid (pH ~2) and the same mixture after the enzymatic modification. The similarity between the spectra indicates that the modified chitosan is primarily chitosan. As shown in Figure 8, both spectra are characterized by a peak at ~2.1 ppm, attributed to the CH_3_ protons of the N-acetylglucosamine residues. In addition, as shown in Figure 8 and Figure 9, the peaks observed at 3.2 and 4.9 ppm correspond to C–2 and C–1 protons of the glucosamine residues. Peaks between 3.4 and 4.0 ppm could be attributed to the C–3, C–4 and C–5 protons, as well as the two C-6 protons. The spectra in Figure 9 show an enlargement of Figure 8 in the region of vinyl and aromatic protons (between 6.0 and 7.0 ppm). A comparison of the spectra in Figure 9 reveals that the enzymatically modified chitosan is characterized by a decrease in the height of the peaks of ferulic acid in the range of 8 ppm, with the simultaneous appearance of a wide absorption range at 7 ppm. The broad peaks appeared between 6.9 and 7.2 ppm in the spectrum of the enzymatically modified chitosan (red), suggesting that it is probably a mixture of various oligomers of ferulic acid. These peaks are absent from the spectrum of the mixture of chitosan and ferulic acid, in the absence of the enzyme laccase. A similar change was observed by Kumar et al. [36] for the enzymatic modification of chitosan with chlorogenic acid. The authors attributed the appearance of the broad peaks between 6.1 and 7.7 ppm in the reaction mixture to the existence of possible various oligomers of chlorogenic acid bound onto the chitosan, in a similar manner to our case, as well. Thus, these findings support the evidence that both the unreacted ferulic acid and the ferulic acid derivatives were covalently grafted onto chitosan.

### 3.7. Estimation of the Grafting Efficiency (Folin)

To access whether the enzymatic modification of the biopolymers with the laccase-oxidized ferulic acid was successful, we evaluated the binding percentage of phenolics according to the Folin–Ciocalteu method [42] by quantifying the amount of total phenolics (mM) bound onto the enzymatically modified CS, CS–GEL and CMC films (Table S1). According to the results presented in Table 1, the modification was successful for all the biopolymers. However, the CS–GEL film exhibited the highest grafting efficiency (95%), followed by CS and CMC, which presented binding efficiencies of 70% and 55%, respectively. These results indicate that due to the heterogeneity of the biopolymers’ nature and structure, different cross-linking reaction mechanisms might occur between the laccase-oxidized ferulic acid and the reactive groups of each polymer, resulting in different binding percentages.

### 3.8. Evaluation of the Antioxidant Activity of the Biopolymers and Their Enzymatically Functionalized Derivatives

#### 3.8.1. Antioxidant Activity According to the Oxidized ABTS Protocol

Since the binding of the phenolic acid derivatives onto the CS, CS–GEL and CMC films proved to be successful, the scavenging effect of the prepared membranes was first studied through the oxidized ABTS protocol (ABTS •+). ABTS, a blue-green color compound, is decolorized in the presence of an antioxidant. Hence, it is an extensively applied protocol for the determination of antioxidant activity. Hence, we examined both the antioxidant activity of the enzymatically modified membranes as well as their individual components, namely, ferulic acid (FA), chitosan (CS), chitosan–gelatin (CS–GEL) and CMC.

**Figure 10:**
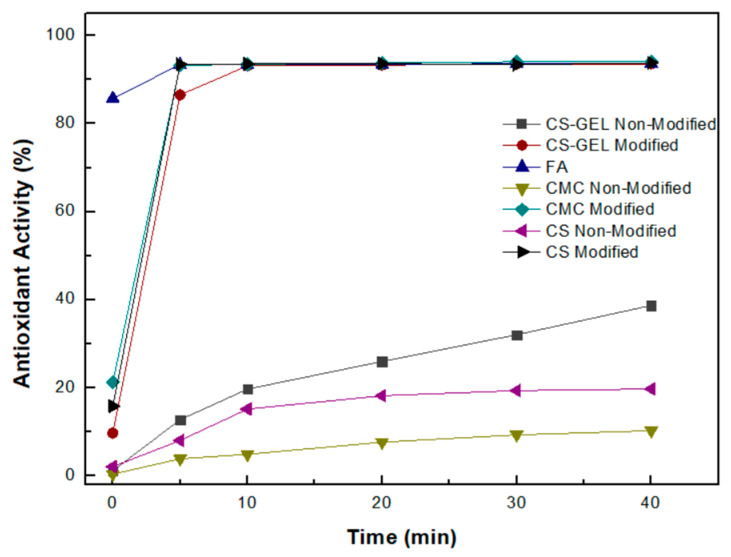
Percentage antioxidant activity of the individual biopolymers and the enzymatically modified biopolymers with ferulic acid, according to the protocol of oxidized ABTS, as a function of time. (The standard deviation was less than 5% in all cases.)

As shown in Figure 10, among the non-modified CS, CMC and CS–GEL membranes at a final concentration of 0.5 mg/mL each, the CS–GEL presented the strongest antioxidant activity (about 40%), followed by the CS and CMC membranes, which exhibited about 15% and 10% antioxidant activity, respectively, after 40 min. The low antioxidant activity of chitosan has been attributed to the inter- and intramolecular hydrogen bonds developed within the chitosan network, as previously reported [43], while the incorporation of gelatin may alter the chemical bonds within the chitosan network, allowing higher accessibility of ABTS to react with the molecule, resulting in a higher antioxidant activity. 

Concerning the enzymatically modified CS, CS–GEL and CMC, as shown in Figure 10, all membranes tested presented strong antioxidant activity, more than 90%, suggesting their successful modification as well as their potential application as effective antioxidants in the food industry. The improved antioxidant activities, also reported in the literature, for the modification of chitosan with ferulic acid and laccase, have been attributed to the probable introduction into the chitosan of a group that acts as an H atom donor formed by the oxidized ferulic derivatives [15,43].

#### 3.8.2. Antioxidant Activity According to the DPPH Protocol

The determination of a system’s radical scavenging activity is a crucial parameter in its characterization due to the role of free radicals in biological systems. The effect of antioxidants on DPPH radical scavenging is attributed to their hydrogen-donating ability [17]. The comparative SC_50_ values of the enzymatically modified and non-modified films are shown in Table 2. Initially, both the non-modified CS and the non-modified CMC showed no reduction in DPPH radicals, probably due to inter- and intramolecular hydrogen bonding, as previously reported in the literature [15,19].

However, the enzymatic modification of all polymers resulted in a significant increase in their antioxidant activity. In the case of CS, a two-fold increase in its antioxidant activity compared with the non-modified CS was observed; for the CS–GEL film, more than a one-and-a-half-fold increase was observed; while in the case of CMC more than a seven-fold increase in its antioxidant activity was observed. These results could be attributed to the successful grafting of high quantities of the oxidized ferulic acid onto the various polymers. The introduction of an H atom-donating group, resulting from the laccase-catalyzed oxidation of FA, seemed effective towards the development of strong antioxidant polymers. These results confirm the successful enzymatic cross-linking of the polymers tested with the products of the laccase-catalyzed oxidation of ferulic acid. Similar results, suggesting a significant increase in DPPH scavenging activity, have been reported for the enzymatic modification of chitosan with ferulic acid and ethyl ferulate [15], as well as for ferulic acid-grafted chitosan using recombinant bacterial laccase [12], also indicating that the antioxidant properties of the grafted chitosan were substantially improved.

### 3.9. Evaluation of the Antimicrobial Activity of the Biopolymers and Their Enzymatically Functionalized Derivatives

**Figure 11:**
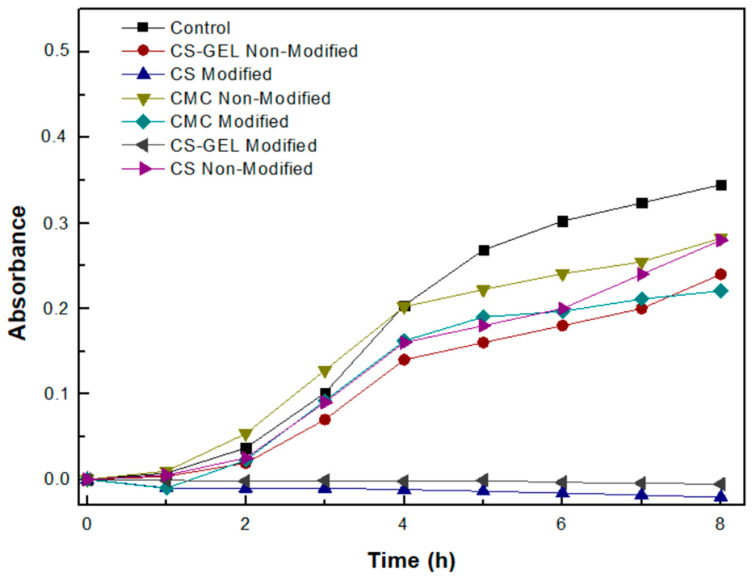
Growth curve of bacterial populations of strain BL21DE3 after 12 h of incubation in the presence of a certain amount of enzymatically modified and non-modified biopolymer membranes as a function of time. (The standard deviation was less than 5% in all cases.)

In addition to antioxidant activity, we also tested the antimicrobial activity of the enzymatically modified membranes. The bacterial strain used was the BL21DE3, which belongs to the Gram-negative (Gram^−^) bacteria. According to the results shown in Figure 11, the non-modified CS and CS–GEL films appear to have antimicrobial activity, attributed to the positively charged amino groups of chitosan that interact with the negatively charged microbial cell membranes, preventing cell growth, as has been mentioned [17,18], while both the modified CS and CS–GEL totally inhibit bacterial cell growth. However, the positive charge of chitosan depends not only on the pH at which the enzymatic modification has taken place, but also on the compounds that have been attached to it. According to the literature, chitosan modified at pH 4.5, compared to modifications at pH 5.5 or 6.5, presents stronger antimicrobial activity against bacteria, such as *E. coli*, which is consistent with the results obtained in the present study. The proposed hypothesis for the antimicrobial action of modified chitosan with phenolic acids argues that the hydroxyl group of the phenolic acid acts as a proton exchanger, reducing the pH gradient along the cytoplasmic membrane and thus inhibiting the transport of calcium and potassium through the calcium channels. As a result, the depletion of ATP supplies leads to cell death [44]. Thus, our results suggest that the successful modification of the CS and CS–GEL films with ferulic acid leads to innovative materials with strong antioxidant activity.

Concerning the antimicrobial activity of the modified and non-modified CMC films, as shown in Figure 10, the non-modified CMC already presents a low antimicrobial activity, which is slightly enhanced after its enzymatic modification with ferulic acid. A significant reduction in bacterial viability for *E. coli* has been also reported in the literature for cellulose membranes developed through the chemical grafting of aminoalkyl groups, an approach intending to mimic the intrinsic antimicrobial properties of chitosan [44]. These results suggest that the antimicrobial activity of various materials could be enhanced through certain modifications. The enhanced antimicrobial activity of the modified CMC membrane could be attributed to modifications induced by the grafting of ferulic acid, resulting in different interactions with the cell membrane, probably inducing irreversible damages to the cytoplasmic membrane and thus inhibiting cell growth [10,41].

## 4. Conclusions

The present work demonstrated, initially, the development and optimization of a bi-enzymatic system comprising a feruloyl esterase and an endo-xylanase for the production of ferulic acid from arabinoxylan. Consequently, a series of various polymers, including chitosan, cellulose and a hybrid chitosan–gelatin polymer, were functionalized with the ferulic acid produced through the action of an oxidoreductase enzyme, namely, laccase from *Agaricus bisporus*. The efficient cross-linking of the biopolymers with the products derived from the laccase-mediated oxidation of ferulic acid was confirmed through various spectroscopic methods, such as UV–Vis spectroscopy, ATR spectroscopy, SEM and NMR. Finally, the enzymatically modified derivatives were characterized by strong antioxidant and antimicrobial properties significantly superior to the biological activities that characterized the non-modified polymers. 

This work highlights the interest in applying environmentally friendly processes for the development of functional biopolymers with enhanced biological activities, opening up new strategies for their application in many biotechnological areas, ranging from food packaging and antioxidant food additives to pharmaceuticals and cosmetics. Our ongoing research also includes the incorporation of immobilized biocatalysts into these processes to ensure the advantage of reusing the biocatalyst for repetitive reaction cycles. 

## Figures and Tables

**Table 1:** Estimation of the phenolics bound onto the enzymatically modified CS, CS–GEL and CMC membranes according to the Folin–Ciocalteu method. (The standard deviation was less than 5% in all cases.)

Sample	Grafting Efficiency (%)
CS	70
CS–GEL	95
CMC	55

**Table 2:** SC_50_ (mg/mL) values for the individual biopolymers and the enzymatically modified biopolymers with ferulic acid, according to the DPPH protocol. (The standard deviation was less than 5% in all cases.)

	CMC Non-Modified	CMC Modified	CS Non-Modified	CS Modified	CS–GEL Non-Modified	CS–GEL Modified
SC_50_ (mg/mL)	75	10	70	35	35	20

## Supplementary Materials

The following supporting information can be downloaded at: https://www.mdpi.com/article/10.3390/biom12070992/s1, Figure S1: HPLC chromatogram; Figure S2: Control experiment; Table S1: Total phenolics (mM) bound onto the enzymatically modified CS, CS–GEL and CMC.
