# Supplementary material for: Development of a Multi-Enzymatic Approach for the Modification of Biopolymers with Ferulic Acid
Source: Biomolecules. 2022 Jul 17;12(7):992. doi: 10.3390/biom12070992 (PMC9312976; doi:10.3390/biom12070992)
Supplement: Supplementary file 1 [file biomolecules-12-00992-s001.zip › biomolecules-1792323-supplementary.pdf]

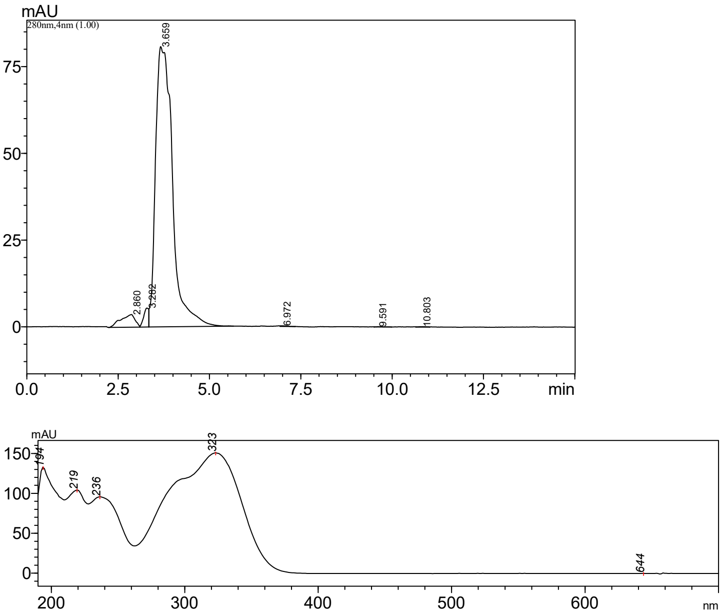

Figure S1. HPLC chromatograph (above) and the UV spectrum (below) of the ferulic acid (Retention time 3.6 min) produced through the synergistic action of xylanase and esterase at a mass ratio 1:5 and arabinoxylan concentration 20 mg/mL.

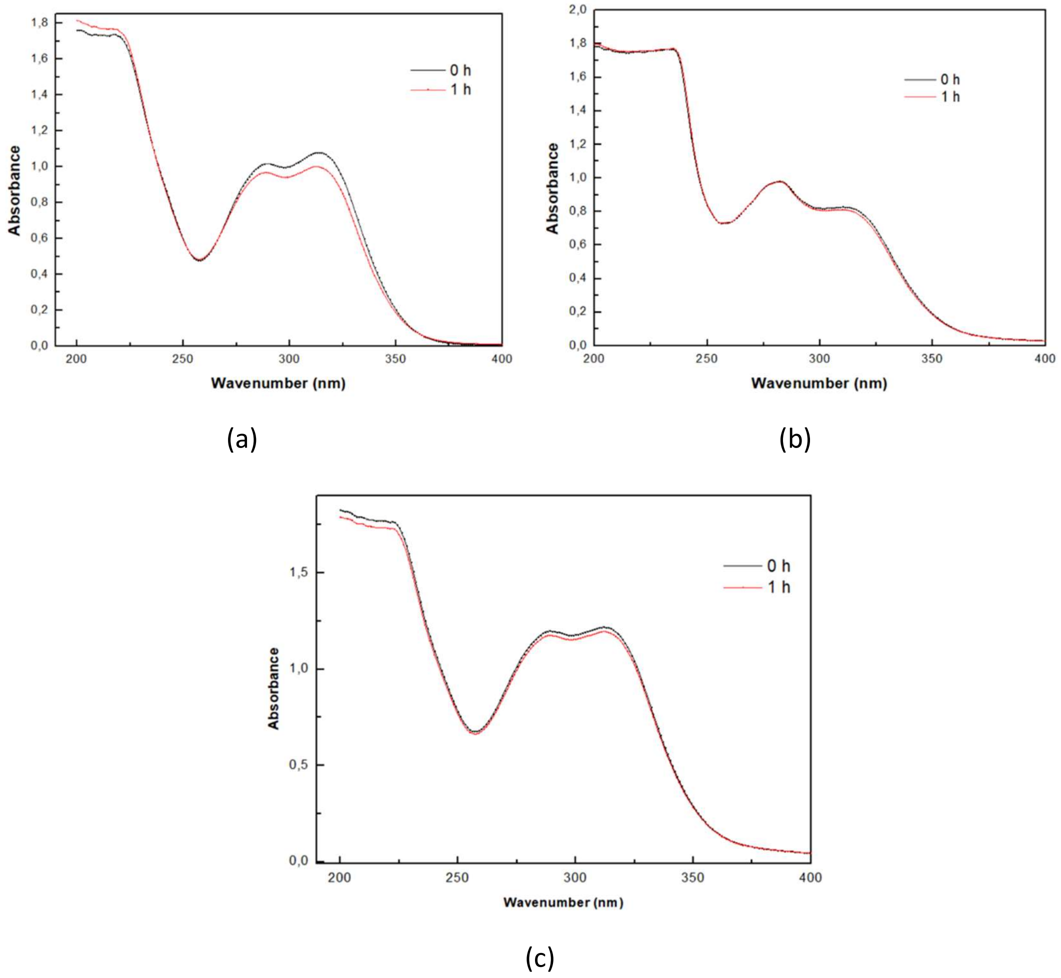

Figure S2. Spectrophotometric monitoring of the control samples (**a**) CMC and ferulic acid solution (**b**) CS-GEL and ferulic acid solution and (**c**) chitosan and ferulic acid solution, in the absence of laccase after 1h of incubation at 30 ^{o}C.

Table S1. Amount of total phenolics (mM) bound onto the enzymatically modified CS, CS-GEL and CMC membranes as estimated by the Folin-Ciocalteu reagent (the standard deviation was less than 5% in all cases).

Sample	Phenolics (mM)
CS	3.50
CS-GEL	4.75
CMC	2.75
